# Myelin Changes in H-ABC Tubulinopathy Revealed by Nile Red Hyperspectral Imaging

**DOI:** 10.3390/ijms27219934

**Published:** 2026-11-06

**Authors:** Carla Jaramillo-Restrepo, Valeria Piazza, Jose R. Eguibar, Carmen Cortes, Victor H. Hernandez

**Affiliations:** 1 Centro de Investigaciones en Óptica A.C., Leon 37150, Guanajuato, Mexico; carlajr@cio.mx; 2 Laboratorio de Neurofisiología de la Conducta y Control Motor, Instituto de Fisiologia, Benemerita Universidad Autonoma de Puebla, Puebla 72000, Puebla, Mexico; jose.eguibar@correo.buap.mx (J.R.E.); carmen.cortes@correo.buap.mx (C.C.); 3 Laboratorio de Neurobiologia Cognitiva, Instituto de Fisiologia, Benemerita Universidad Autonoma de Puebla, Puebla 72000, Puebla, Mexico; 4 Departamento de Ingenierias Quimica, Electronica y Biomedica, Division de Ciencias e Ingenierias, Universidad de Guanajuato, Leon 37150, Guanajuato, Mexico

**Keywords:** hyperspectral imaging, Nile Red, myelin, H-ABC tubulinopathy, *taiep* rat, spectral phasor analysis

## Abstract

Hypomyelination with atrophy of the basal ganglia and cerebellum (H-ABC) is a recently described tubulinopathy caused by mutations in the tubulin beta-4a isotype. It is a severe disorder characterized by progressive myelin loss and neurological impairment. The *taiep* rat, a spontaneous TUBB4A mutant, is a model for investigating the cellular and molecular mechanisms underlying this disease. Homozygous *taiep* rats exhibit hallmark features of H-ABC, including early-onset hypomyelination, progressive demyelination, and microtubule accumulation in oligodendrocytes, whereas heterozygous animals appear to maintain normal myelin structure using conventional techniques. We combined hyperspectral imaging (HSI) and Nile Red (NR) staining to differentiate homozygous *taiep* and wild-type animals and determine whether subtle myelin alterations could be detected in heterozygous rats. Remarkably, heterozygous animals also showed changes in the Nile Red emission profile, with fluorescence shifted toward longer wavelengths compared with controls, uncovering previously unrecognized modifications in the myelin microenvironment. Using spectral phasor analysis, these alterations were localized and quantified without model assumptions, demonstrating the sensitivity of this approach for detecting subtle pathological changes. Together, our findings contribute to the characterization of myelin-associated spectral changes in this tubulinopathy and support the use of spectral microscopy to distinguish alterations in myelin properties, with potential applications in the study of other white matter disorders.

## 1. Introduction

Tubulinopathies represent a group of neurodegenerative disorders resulting from mutations in genes that encode tubulin, leading to disruptions in the function of microtubules, essential components of the cytoskeleton [1]. Even though tubulin isotypes are widely expressed across tissues, their critical role in brain development makes the nervous system particularly susceptible to the consequences of these mutations [2]. Clinically, tubulinopathies manifest as cerebral malformations and neurodevelopmental or neurodegenerative syndromes, primarily impairing neuronal generation, migration, and cortical organization [3,4]. Within this group, mutations in *TUBB4A* are peculiar for their unique association with white matter abnormalities, specifically hypomyelination and myelin loss [5,6]. One notable example is hypomyelination with atrophy of the basal ganglia and cerebellum (H-ABC), a severe disorder marked by defective myelin production, progressive atrophy of critical brain regions, and a broad range of neurological symptoms, including spasticity, ataxia, and cognitive impairments [6].

We have demonstrated that the *taiep* rat, a spontaneous *TUBB4A* mutant originating from the Sprague–Dawley strain [7], carries the *TUBB4A* A302T mutation and represents a valuable animal model for studying tubulinopathies [8,9]. This rodent exhibit key pathological hallmarks, including hypomyelination at birth and progressive central nervous system (CNS) demyelination [10], closely mirroring the disease course of patients with H-ABC [8]. Consistent findings from magnetic resonance imaging (MRI) and histopathological analyses further demonstrate extensive demyelination [11] and abnormal microtubule accumulation within oligodendrocytes [10], which are characteristic of this tubulinopathy.

In *taiep* rats, hypomyelination is already evident at 2–3 months, affecting both the spinal cord and brain. In the spinal cord, axons across all columns appear thinly myelinated or unmyelinated, with the dorsal column being most affected; while some large-diameter axons retain normal myelin thickness, tracts composed mainly of small-caliber axons, such as the fasciculus gracilis and corticospinal tracts, are uniformly hypomyelinated. At the same age, reduced myelin is also observed in major brain white matter tracts, including the cerebellar medulla, folia, and corpus callosum, with histological staining confirming markedly less myelin in the brain stem and forebrain compared to controls. These alterations progress to an almost complete loss of myelin by 12 months [12,13].

In contrast, heterozygous *taiep* rats show no detectable myelin abnormalities in the spinal cord, even at 16 months of age. Histochemical staining with nitroblue tetrazolium has confirmed that their myelin remains structurally intact [12]. This raises the question of whether more sensitive approaches could uncover alterations in myelin that escape conventional methods. In this context, we aimed to evaluate whether hyperspectral microscopy using Nile Red (NR), a dye with strong affinity for myelin [14], could detect early or incipient changes in these animals. For this purpose, we examined 9-month-old heterozygous rats, using the same age as in experiments with *taiep* rats.

The results revealed a red-shift in the emission spectrum of NR compared to control rats, consistent with subtle modifications in the myelin microenvironment—an observation reported here for the first time. These findings were derived from hyperspectral imaging (HSI), a technique that captures the complete emission spectrum at each pixel of the image, thereby providing both spatial and spectral information simultaneously [15]. To analyze this complex dataset, we applied the spectral phasor approach, which transforms spectral information into a pair of coordinates, (1) and (2), in a phasor plot [16]. This mathematical method not only simplifies high-dimensional spectral data without the need for pre-assumptions or fitting models, but also enables the identification of localized alterations within the tissue. Together, our results highlight the potential of spectral microscopy as a powerful, label-based strategy for highly sensitive detection of subtle myelin alterations, including changes that may precede overt structural abnormalities.

## 2. Results

### 2.1. Solvatochromic Emission Shift of Nile Red Across Solvents

To explore the environmental sensitivity of Nile Red (NR), we examined its fluorescence behavior in solvents of varying polarity. When dissolved at a concentration of 20 µM and excited at 488 nm, NR displayed clear shifts in emission depending on the solvent. In non-polar solvents such as hexane, NR emitted at shorter wavelengths (peak at ~560 nm green), while in polar solvents like acetone, the emission was red-shifted (~640 nm) (Figure 1A). The normalized spectra in (Figure 1B) reveal a substantial shift of approximately 80 nm, indicating that the emission spectra of NR respond to chemical changes in the surrounding environment.

Although the spectral red-shift of NR generally follows solvent polarity, the trend is not strictly determined by polarity alone. For instance, despite acetonitrile being more polar than acetone, the emission maximum in acetone is shifted further to the red. This suggests that additional solvent parameters—such as hydrogen-bonding capacity, polarizability, and specific solute–solvent interactions—also contribute to the solvatochromic response. Nonetheless, the key outcome of these findings is that the consistent solvatochromism observed across solvents provides a robust basis to extend our experiments into more heterogeneous and biologically relevant systems, such as tissue, where multiple microenvironmental factors coexist.

### 2.2. Nile Red for Detecting Myelin Alterations in Tissue

We next investigated whether the solvatochromism of NR, previously observed in Figure 1, could also be detected in specific brain regions of the *taiep* rat model. Figure 2A presents spectral confocal micrographs of fixed brain cryosections from control and *taiep* rats, incubated for 10 min in an NR solution (20 µM). The region of interest was the corpus callosum (CC), a structure rich in myelin, where differences between healthy and altered conditions can be expected. The true-color images, generated to reproduce the visual appearance under a conventional fluorescence microscope, showed brightly stained, uniformly orange areas in both control and *taiep* samples. However, pseudocolored images in which each pixel was assigned a color according to its emission spectrum, revealed morphological and biochemical differences. In control rats, the CC exhibited a predominant yellow hue, which we assign to a “healthy” condition on the color scale. In contrast, the CC of *taiep* rats displayed a marked spectral shift toward violet, reflecting an altered biochemical environment consistent with myelin degeneration.

Phasor plot analysis (Figure 2B,C) revealed a clear spectral separation between the CC of control and *taiep* rats. The applied color scale represents the phase angle of each pixel’s emission spectrum, ranging from 30° (490 nm) to 180° (727 nm), corresponding to the spectral center of mass. In control samples (Figure 2B), the phasor coordinates formed a compact cluster within the 514–550 nm range, indicating a relatively homogeneous emission profile consistent with the lipid-rich, compact myelin of healthy tissue. In contrast, the *taiep* CC (Figure 2C) showed more dispersed phasor distribution and pronounced shift toward 600–680 nm, reflecting a redder emission component. This displacement was confirmed in the spectral emission curves (Figure 2F), where the average emission peak in the control rat was 579 ± 2 nm, while in *taiep* rats it shifted to 615 ± 2 nm, representing a 36 nm red-shift, indicative of changes in the myelin microenvironment. Such spectral modifications may reflect alterations in lipid packing and local polarity within the myelin sheath [14,17], consistent with the less compact, thinner myelin reported in the *taiep* rat [10,12].

Beyond the spectral center of mass, phasor analysis allows the extraction of additional parameters such as eccentricity and radial standard deviation, which describe the directional trends and overall spread of the spectral distribution. These metrics revealed striking differences between conditions: in the control rat (Figure 2B), the phasor points were tightly clustered around a main component at 534 nm, consistent with a uniform emission profile. In contrast, the *taiep* distribution was broader and more dispersed, extending from ~600 to 680 nm, indicative of a wider range of spectral components. Quantitative analysis confirmed significantly higher eccentricity and radial standard deviation values in the *taiep* rat (Figure 2D,E), reflecting greater spectral heterogeneity. Such heterogeneity is consistent with alterations in the biochemical environment of myelin, likely arising from structural disorganization and changes in lipid composition [14].

The modulation analysis revealed values of 0.52 ± 0.03 for control and 0.69 ± 0.09 for *taiep* (mean ± SEM, respectively), suggesting an apparent increase in modulation in the *taiep* CC. Under typical conditions, modulation is inversely related to spectral width, with higher values reflecting narrower emission profiles. However, despite the broader spectral profile observed in *taiep* compared with the control, modulation remained higher. This apparent discrepancy may arise from a more asymmetric spectral broadening in *taiep*, in which the main emission peak retains a high relative amplitude while additional shoulders extend toward longer wavelengths. Such a distribution can preserve higher phasor modulation values despite increased overall spectral spread, resulting in the *taiep* phasor coordinates plotting farther from the origin than those of the control. Together, these results demonstrate that within our experimental system using the *taiep* rat, hyperspectral imaging of NR solvatochromism, combined with phasor-based spectral analysis, reliably detects both spatial and biochemical alterations in myelin, providing a sensitive metric to distinguish healthy from affected tissue.

### 2.3. Spectral Microscopy Uncovers Subtle Myelin Changes in Heterozygous Rats

After establishing that NR reliably detects myelin alterations in homozygous rats with H-ABC tubulinopathy, we next asked whether spectral microscopy using this dye would be sensitive enough to identify subtler changes in heterozygous (_A302T_/^+^) rats, if present. All previous experiments had been performed in homozygous animals, which display clear pathological and clinical features, but heterozygous rats do not exhibit overt clinical manifestations [12]. Nevertheless, previous reports indicate that heterozygous transgenic mice carrying the most frequent H-ABC mutation (_D249N_/^+^) exhibit hypomyelination [18]. This raised the possibility that our heterozygous rat model might also exhibit subtle myelin changes that are difficult to observe by clinical signs or conventional histological approaches.

To test this hypothesis, we obtained coronal brain sections from 9-month-old heterozygous rats and stained them with NR under the same conditions (Figure 3A top) used in homozygous animals (previous experiments). Spectral analyses revealed a small but consistent red shift in the NR emission spectrum, likely due to changes in the myelin microenvironment (Figure 3C). This is further supported by the pseudocolor image (Figure 3A bottom), where the white matter exhibits a broader spectral distribution, appearing predominantly green with localized bluish regions, which—according to the color scale—corresponds to an altered condition. A similar pattern is observed in the pseudocolored images of larger brain sections (Figure S1). The phasor distribution for the heterozygous condition (Figure 3B; corresponding to the top panel in Figure 3A) spanned a wavelength range of 545–587 nm, indicating a slight displacement toward longer wavelengths. In contrast, spectral analysis (Figure 3C), which reflects the mean emission across all analyzed cases, showed a peak centered at 589 nm. This represents an approximate ~10 nm red shift compared with wild-type controls (579 nm) and places the heterozygous condition intermediate between control and homozygous *taiep* rats (615 nm). This positioning supports the notion that the heterozygous condition exhibits myelin alterations, despite the absence of overt clinical signs.

Phasor plot analysis of the CC across the three groups (Figure 3D) further confirmed these findings, clearly separating WT, heterozygous, and homozygous *taiep* rats. Although the centers of mass of WT and _A302T_/^+^ phasors remained close to each other, both were distinctly separated from the *taiep* rat. The heterozygous group displayed a measurable displacement toward the *taiep* cluster, consistent with the ~10 nm red shift observed in the spectral emission curves between controls and heterozygotes, indicative of subtle but detectable changes in the myelin microenvironment.

Quantitative analysis of phasor parameters revealed a significant increase in eccentricity and radial standard deviation in both heterozygous and homozygous animals compared with controls (Figure 3E,F), with heterozygotes again showing intermediate values. These changes indicate increased heterogeneity and altered organization of the spectral phasor distributions. Notably, although the geometry and spread of the phasor clouds varied among individual *taiep* samples, their central spectral regions remained comparatively consistent, indicating that local spectral heterogeneity can vary without a proportional displacement of the dominant spectral population. Together, these findings highlight the sensitivity of spectral phasor analysis to detect subtle microstructural and biochemical alterations in the myelin environment that would remain undetected by standard histological approaches.

## 3. Discussion

In this study, we evaluated the use of Nile Red (NR) combined with spectral microscopy as a sensitive approach to investigate myelin alterations in H-ABC tubulinopathy as well as in other leukodystrophies [14]. Methods for monitoring subtle changes in tissue lipid chemistry remain limited, particularly if preservation of tissue and high-resolution imaging of cellular details are required [19]. Nile Red, a well-established lipophilic dye, has long been employed to visualize cell membranes and other lipid-enriched domains [20,21,22] and has even been applied in studies of living organisms [23]. A distinctive property of this fluorophore is its solvatochromism, through which the emission spectrum changes in response to the surrounding environment. Additionally, it interacts strongly with anisotropic media such as nematic liquid crystals, which modulate its absorption and fluorescence spectra [24]. Considering the lipid-rich composition and liquid crystalline properties of myelin [25], we reasoned that a lipophilic solvatochromic probe like NR would not only serve as a valuable reporter of the physicochemical properties of myelin sheaths but also as a highly reliable marker to reveal fine morphological features with elevated spatial resolution.

Previous studies have shown that NR fluorescence intensity decreases and its emission shifts toward longer wavelengths in more polar environments. This happens because, during the S_0_ → S_1_ transition, the molecule undergoes a substantial alteration in its dipole moment, enhancing its interaction with polar solvents and resulting in a red-shifted emission [14]. However, as shown in our experiments (Figure 1B), the solvatochromic response of NR does not follow a strictly linear relationship with solvent polarity. For instance, the emission of NR is more red-shifted in acetone than in acetonitrile, even though acetonitrile is known to be more polar. This suggests that, beyond polarity, other physicochemical factors, such as hydrogen bonding, the ability of the solvent to polarize, and unique interactions between solute and solvent, also contribute to the observed spectral shifts.

Conventional fluorescent images of NR-labeled brain slices showed a uniform color across the three conditions analyzed—WT, _A302T_/^+^, and homozygous *taiep* rats—with no visible differences. In contrast, spectral analysis uncovered significant changes in the emission spectrum, with average peaks of 579 ± 2 nm for WT, 589 ± 3 nm for _A302T_/^+^, and 615 ± 2 nm for homozygous *taiep* rats. The ~36 nm red shift observed between the control group and homozygous *taiep* rats can be attributed, among other factors, to a more polar interaction environment for NR, resulting from less compact myelin [14,26]. This structural alteration is consistent with the significant increase in the g-ratio and the presence of progressively thinner myelin sheaths previously described in the *taiep* rat [10]. MRI studies have also demonstrated that *taiep* rats exhibit hypomyelination followed by progressive demyelination in several central tracts, including the corpus callosum and the anterior commissure [11]. Notably, in heterozygous rats—which do not show evident myelin alterations or clinical manifestations of the disease—a red shift of approximately 10 nm in the NR emission spectrum was detected. The variability observed in this group likely reflects heterogeneity within tissue that is largely considered “apparently healthy,” where subtle signs of myelin alterations may already be emerging. These findings suggest that NR spectral analysis is sensitive to subtle alterations in myelin-associated spectral properties that may not be readily apparent through conventional observation, supporting its potential as a complementary approach for characterizing myelin modifications.

Importantly, the sensitivity of NR-based spectral analysis to changes in the myelin microenvironment is not restricted to the *taiep* model. Teo et al. [14] demonstrated that NR fluorescence spectroscopy detects physicochemical alterations in histologically intact myelin during the early stages of cuprizone-induced demyelination and in normal-appearing white matter from human multiple sclerosis (MS) brains, indicating that spectral changes can precede overt structural myelin loss detectable by conventional histological methods. In the cuprizone model, significant changes toward a more polar myelin environment were detected after only 2 days of treatment, when Luxol Fast Blue staining did not yet reveal detectable demyelination; similar observations were reported by Jhelum et al. [27]. Beyond demyelination, NR spectral microscopy has also been used to follow biochemical changes occurring during peripheral nerve remyelination, demonstrating that the emission spectrum evolves with myelin maturation both in vitro and in vivo [28]. More recently, NR spectroscopy has been shown to resolve differences in the physicochemical properties of myelin across CNS and PNS regions, developmental and biological conditions, and live ex vivo white-matter preparations [17]. The broader applicability of this approach is further supported by spectral-phasor studies showing that NR can quantitatively map membrane micropolarity and distinguish heterogeneous lipid environments in living cells and tissues, as well as detect membrane-microenvironment alterations associated with amyloid–membrane interactions [29]. Together, these studies support the use of NR-based spectral imaging as a sensitive reporter of subtle physicochemical changes in lipid-rich membranes and strengthen the interpretation that the spectral alterations observed here reflect changes in the myelin microenvironment rather than a phenomenon specific to the *taiep* model.

These results fit within a broader landscape of imaging innovations that are reshaping how myelin-related disorders are studied. The development of modern imaging technologies has significantly advanced this field. While multiphoton microscopy and FLIM have proven valuable for quantitative evaluation of tissue, their application is often limited by cost and technical requirements. Hyperspectral imaging (HSI), on the other hand, is increasingly available in commercial confocal systems that operate with single-photon excitation. Unlike other techniques, HSI enables the analysis of spectral information without assuming the identity of the fluorescent species present. This feature makes it particularly suitable for exploring pathological changes in myelin, providing a practical and evidence-supported strategy for studying disease models.

Nonetheless, analyzing HSI data has challenges due to its high dimensionality and complexity. To address this, a mathematical approach known as the phasor transform can be applied. This method converts multidimensional data into complex numbers, represented by the *G* and *S* coordinates (x and y, respectively), thereby enabling a straightforward visualization and comparison of spectral signatures.

In our study, conventional fluorescence images of the corpus callosum stained with NR under the three experimental conditions revealed a uniform orange signal, with no evident differences. However, applying spectral analysis transformed the interpretation. Through the phasor transform, each pixel was pseudocolored according to its spectral shape, making a marked shift visible. In the *taiep* rat (Figure 2A), the pseudocolor map revealed a predominance of violet tones, indicating an altered condition, as indicated by the color bar. Spectral phasor analysis of the HSI data further confirmed this shift, showing that *taiep* rats displayed an increased phase component, corresponding to longer-wavelength fluorescence, compared with control rats. This can be explained by an increase in membrane polarity, a phenomenon that has been associated with structural loosening of the lipid bilayer and reduced lipid packing during myelin disruption [17,26]. Other studies using solvatochromic dyes such as Laurdan and NR have reported that demyelination processes are accompanied by higher polarity and hydration of the myelin sheath, reflecting alterations in lipid composition, increased water penetration, and decreased compactness of the multilamellar structure [14,30,31].

By contrast, in control rats, the lower polarity likely reflects the compact nature of myelin, but it may also be influenced by age-related changes in white matter and myelin, which are well-documented phenomena [32,33]. For instance, studies have shown that the medial corpus callosum in male mice displays age-dependent changes in polarity, with significantly lower polarity observed at 31 weeks compared to 12 weeks of age. Since our experiments were conducted in rats aged 9–10 months, it is reasonable to consider that similar age-related processes might be occurring in all genotypes studied. This raises important questions: could the spectral behavior of NR vary at different stages of development or aging in control and *taiep* rats? And does the interaction of NR with myelin lipids change significantly across time? Addressing these questions will require further experimental investigation, such as a time-course spectral imaging study in both rats across defined ages and lipidomic profiling of myelin during these stages.

By pointing toward these future directions, our study underscores the power of combining HSI with spectral phasor analysis to uncover early alterations in myelin in H-ABC tubulinopathy. Notably, we report a pioneering observation in heterozygous rats, where subtle myelin changes—undetectable with conventional methods, and therefore not characterized so far—begin to emerge. Beyond its potential application in conditions associated with hypo- or demyelination, this approach may also provide complementary information about differences in myelin-associated properties across developmental or pathological states. By revealing spectral variations related to myelin organization/composition, it could contribute to the characterization of processes involved in myelin formation and alteration.

## 4. Materials and Methods

### 4.1. Preparation of Nile Red Solvent Mixtures for Spectral Acquisition

To record the emission spectra of Nile Red (NR) in different solvent environments, solutions of the dye at 20 µM were loaded into thin glass capillaries (1 mm × 75 mm) by passive capillary filling. To prevent leakage or evaporation, both ends of each capillary were carefully sealed with liquid silicone. Spectral imaging was performed using a Zeiss LSM 710 confocal microscope (Zeiss, Jena, Germany) equipped with a 25X/0.8 LCI Plan-Neofluar immersion objective (Zeiss, Jena, Germany). The samples were excited with a 488 nm laser. For NR diluted in phosphate-buffered saline (PBS), which exhibits reduced fluorescence efficiency, a higher concentration (60 µM) was used to ensure sufficient signal intensity for analysis.

### 4.2. Animals

A total of 12 male rats aged 9–10 months were included in the study, with four animals in each of three experimental groups: wild-type (WT), homozygous *taiep*, and heterozygous *taiep***.** These three experimental groups were used to evaluate the sensitivity of the proposed method for detecting physicochemical alterations in myelin lipids. For this purpose, the corpus callosum was selected because of its high myelin content. In addition, at 9–10 months of age, *taiep* rats exhibit extensive demyelination, white matter atrophy, and ventricular enlargement, making this age and brain region particularly suitable for distinguishing intact from altered myelin [7].

*Taiep* tubulin-mutant rats on a Sprague–Dawley background and healthy control rats (RRID: MGI_5648275) were bred and housed at the Institute of Physiology, Benemérita Universidad Autónoma de Puebla. Heterozygous *taiep* rats were obtained by crossing homozygous taiep males with homozygous wild-type (WT) females. Because the taiep mutation is inherited as an autosomal recessive trait [7], all F1 offspring from these crosses were heterozygous.

Animals were maintained under standard laboratory conditions at a temperature of 22 ± 1 °C and a relative humidity of 30–45%. Rats were housed in transparent acrylic cages (60 × 40 × 17 cm) containing sterile wood-chip bedding (Aspen Chip, Warrensburg, NY, USA). Animals had ad libitum access to a standard laboratory diet (LabDiet 5001, Arizona Feed Country Store, Tucson, AZ, USA); 4.5% fat, 23% protein, 34.43% carbohydrates, and 4.07 kcal/g) and purified water. A 12:12-h light–dark cycle was maintained, with lights on at 07:00. Neither the *taiep* rats nor the healthy controls exhibited additional pathologies unrelated to the experimental phenotype.

All procedures complied with the regulations outlined in the Seventh Title of the Regulations of the General Law of Health regarding Health Research of the Mexican Government (CIBIUG-P19–2019) and adhered to the recommendations of the National Institutes of Health Guide for the Care and Use of Experimental Animals, as well as the NIH and ARRIVE 2.0 guidelines. The work was approved by the “Comité Institucional para el Cuidado y Uso de los Animales de Laboratorio” of the Vicerrectoría de Investigación y Estudios de Posgrado at the Benemérita Universidad Autónoma de Puebla, with approval code VIEP/DI/0058/2025 and approval date 24 February 2025.

### 4.3. Tissue Preparation and Staining

Paraformaldehyde-fixed brain samples kept stored in the institutional tissue bank were embedded in tissue-freezing medium (Leica, 14020108926), and sectioned at 30 µm using a Leica CM1520 cryostat (Leica Biosystems, Nussloch, Germany). Sections were mounted on glass slides and stored at −20 °C.

For Nile Red (NR) staining, a 6 mM stock solution of NR (RRID: SCR_016151) was prepared in DMSO and stored at −20 °C. A 20 µM working solution was freshly prepared in 1× PBS. The staining conditions were selected based on previously reported protocols for Nile Red spectral analysis of myelin [14]. Fixed tissue sections were incubated with NR for 10 min, followed by a single 5-min rinse in abundant PBS. The stained sections were then mounted in PBS on glass microscope coverslips and imaged using a Zeiss LSM 710 confocal microscope (RRID: SCR_013672) (Zeiss, Jena, Germany) equipped with an LCI Plan-Neofluar 25×/0.8 Oil Korr M27 immersion objective (Zeiss, Jena, Germany).

### 4.4. Spectral Image Acquisition and Processing

Hyperspectral fluorescence imaging was performed using a Zeiss LSM 710 confocal laser scanning microscope equipped with a spectral detector. Excitation was carried out using a 488 nm laser at 2% laser intensity. The spectral detector was configured to capture 71 channels covering a wavelength range from 498 nm to 724 nm, with a spectral resolution of 3.2 nm. Fluorescence intensity was extracted directly from each image using the software tools provided by the Zeiss microscopy system (black version). The spectral data were then linearly interpolated to generate vectors of 700 evenly spaced data points, enabling standardized comparison and processing across datasets. Images were acquired at a resolution of 1024 pixels, with a pixel size of 332 nm × 332 nm, using an 8-bit dynamic range. A scan speed setting of 3 was used, corresponding to a pixel dwell time of 6.30 μs.

For HSI analysis, the phasor transform was applied to spectral data. In an HSI stack, each pixel contains an emission intensity spectrum *I*(*λ*) as a function of wavelength (λ) [34,35]. The phasor transform converts *I*(*λ*) into a phasor—a complex number that represents the spectrum as a sinusoidal function characterized by a specific phase and modulation. This transformation allows each spectrum to be represented as a single point in the phasor plot, with Cartesian coordinates *G* and *S*, as defined by the following equations:

(1) $$x=G\left(\lambda \right)=\frac{\int_{\lambda_{min}}^{\lambda_{max}}I\left(\lambda \right)cos\;\left(\frac{2\pi n(\lambda -\lambda_{min})}{\lambda_{max}-\lambda_{min}}\right)\;d\lambda }{\int_{\lambda min}^{\lambda max}I\left(\lambda \right)d\lambda }$$

(2) $$y=S\left(\lambda \right)=\frac{\int_{\lambda_{min}}^{\lambda_{max}}I\left(\lambda \right)sin\;\left(\frac{2\pi n(\lambda -\lambda_{min})}{\lambda_{max}-\lambda_{min}}\right)\;d\lambda }{\int_{\lambda min}^{\lambda max}I\left(\lambda \right)d\lambda }$$

where *I*(*λ*) represents the fluorescence intensity at each wavelength λ, and λ (min) and λ (max) define the lower and upper bounds of the spectral range. The variable *n* refers to the harmonic number used in the phasor transform; in this study, the first harmonic (*n* = 1) was applied.

### 4.5. Spectral Phasor Analysis

Hyperspectral images from tissue samples were analyzed using spectral phasor transformation implemented in Python (version 3.12) [36,37]. After loading the images with the tifffile library, the spectral profile of each pixel was treated as a signal along the wavelength dimension. Phasor coordinates were then computed using the phasor from signal function from the PhasorPy package (version 0.12), which applies the first harmonic (*n* = 1) of the Fourier transform to extract the real (*G*) and imaginary (*S*) components. These components were used to generate two-dimensional phasor plots, offering a compact representation of the spectral content at each pixel.

A 3 × 3 median filter was applied to the real and imaginary phasor values to reduce pixel-level noise. Subsequently, regions with low signal were excluded through intensity-based thresholding (mean intensity > 1). The filtered phasor coordinates were then visualized as a two-dimensional histogram, with the *nipy_spectral* colormap indicating the density distribution across the spectral space.

From the phasor coordinates, the phase (α) and modulation (M) were computed as:

(3) $$\alpha =\tan^{-1}\frac{S}{G}\;;\;M=\;\sqrt{G^{2}+\;S^{2}}$$

where α is related to the spectral center of mass, while the modulation (M) reflects the spectral width and contains information about the full width at half maximum (FWHM). A broader spectrum—i.e., a larger FWHM—yields a lower modulation value, shifting the phasor coordinates closer to the origin. In parallel, a spectral shift toward longer wavelengths (red-shift) results in a phase shift along the counterclockwise direction from the (1,0) point on the phasor plot.

To characterize the overall distribution of phasor coordinates, the center of mass of the phasor cloud was computed as the mean of the real and imaginary components across all pixels. The radial standard deviation was then calculated as the standard deviation of the Euclidean distances from each phasor point to this center of mass, reflecting the degree of spectral dispersion around the mean. In addition, the eccentricity of the phasor distribution was estimated using the covariance matrix of the (*G*, *S*) coordinates. This measure provides a quantitative assessment of spectral heterogeneity: high eccentricity values indicate an elongated distribution, while low eccentricity values correspond to a more homogeneous signal.

To support spectral interpretation, a pseudo-color rendering scheme was implemented by mapping the phase angle of each phasor point to a corresponding hue on a circular HSV color scale. The phase captures the spectral center of mass for each pixel, and it is then used to assign color. A narrow annulus centered at the origin was overlaid on the phasor plot, where each angular wedge (1° increments from 30° to 180°) was colored according to its corresponding hue in the HSV color map. The hue wheel was rotated by 90° counterclockwise so that α ≈ 30° (∼490 nm) was assigned to cyan and α ≈ 180° (∼727 nm) was rendered reddish-orange, consistent with the experimental wavelength range. Wavelength annotations were placed at 30°, 90°, and 180° positions relative to the rotated frame. Additionally, the center of mass of the phasor distribution was marked with a black cross, and dispersion metrics—radial standard deviation and eccentricity—were displayed in an inset for quick visual assessment.

### 4.6. Statistical Analysis

To evaluate differences in spectral and phasor-derived parameters among the experimental groups, a total of 80 images per condition were analyzed for wild-type, heterozygous, and *taiep* rats. These images were obtained from four different animals per group. For each animal, five images were acquired from each tissue across four repeated measurements, yielding a total of 20 images per animal and 80 images per condition. To avoid pseudoreplication, the measurements obtained from each animal were averaged to generate a single representative value per animal for each analyzed parameter. Accordingly, the statistical analysis was performed using one independent value per animal (*n* = 4 animals per group), rather than treating individual images as independent biological replicates. This sample size is reflected in the degrees of freedom reported with the ANOVA F statistics and is explicitly indicated in the corresponding figure captions. The analyzed parameters included the maximum emission wavelength, the standard deviation of the radial component (STD radial), eccentricity, and modulation.

Statistical analyses were performed using R (R Core Team version 4.3.2). Differences among groups were assessed using one-way analysis of variance (ANOVA), followed by Tukey’s post hoc test for multiple comparisons. Prior to statistical inference, the assumptions of ANOVA were evaluated. Normality was assessed using the Shapiro–Wilk test together with examination of the model residuals, while homogeneity of variances was assessed using Levene’s test. No substantial violations of the ANOVA assumptions were detected. Statistical significance was set at *p* < 0.001. Unless otherwise indicated, data are presented as the mean ± standard error of the mean (SEM).

## Figures and Tables

**Figure 1:**
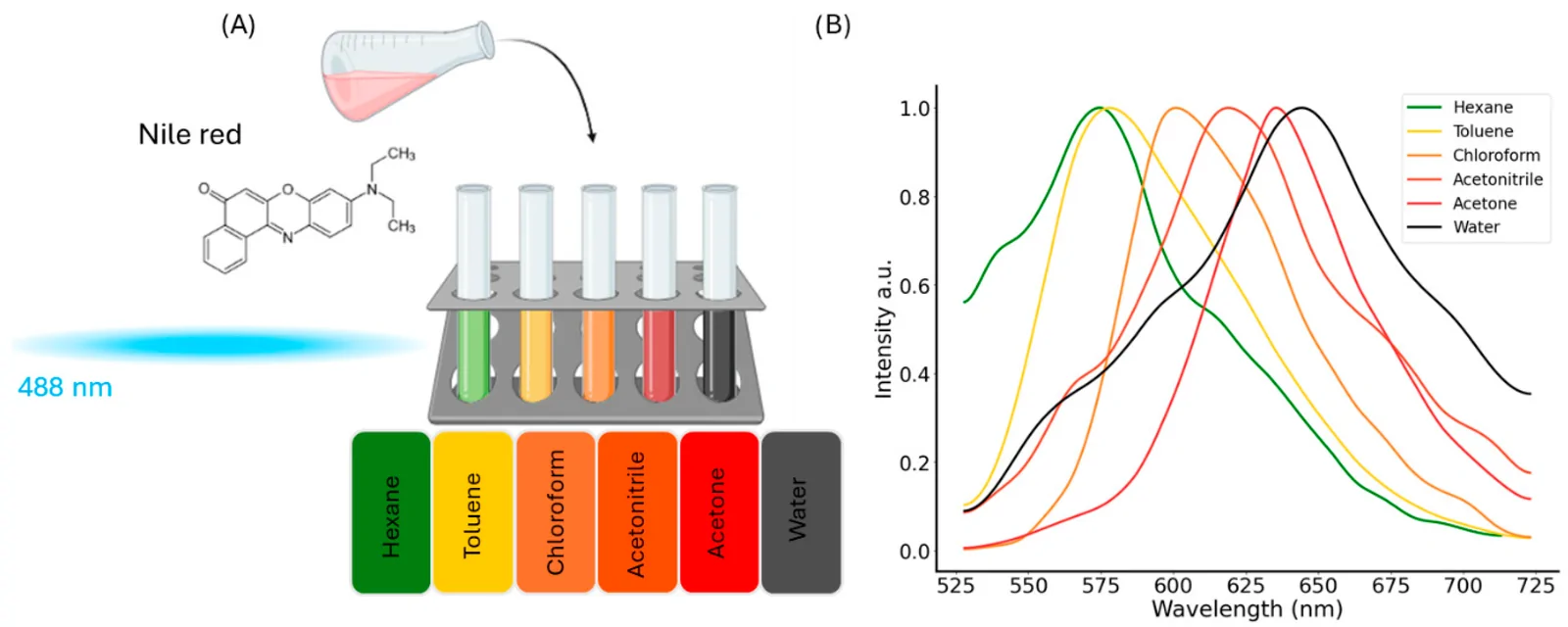
Solvatochromic behavior of Nile Red in solvents of varying polarity. (**A**) Illustration of Nile Red (NR)’s molecular structure and its dissolution (20 µM) in solvents spanning a wide polarity range: hexane, toluene, chloroform, acetonitrile, acetone, and water. Created in BioRender. Restrepo, C. (2026) https://BioRender.com/2xbpajx (accessed on 15 February 2026). The visual color differences in the vials reflect the shift in fluorescence emission as solvent polarity increases. (**B**) Normalized fluorescence emission spectra of NR upon excitation at 488 nm. In non-polar solvents such as hexane, NR exhibits a blue-shifted emission in the green region, whereas in highly polar solvents like acetone and water, the emission undergoes a pronounced red-shift. The data correspond to the mean of three independent experiments, each conducted with three replicates.

**Figure 2:**
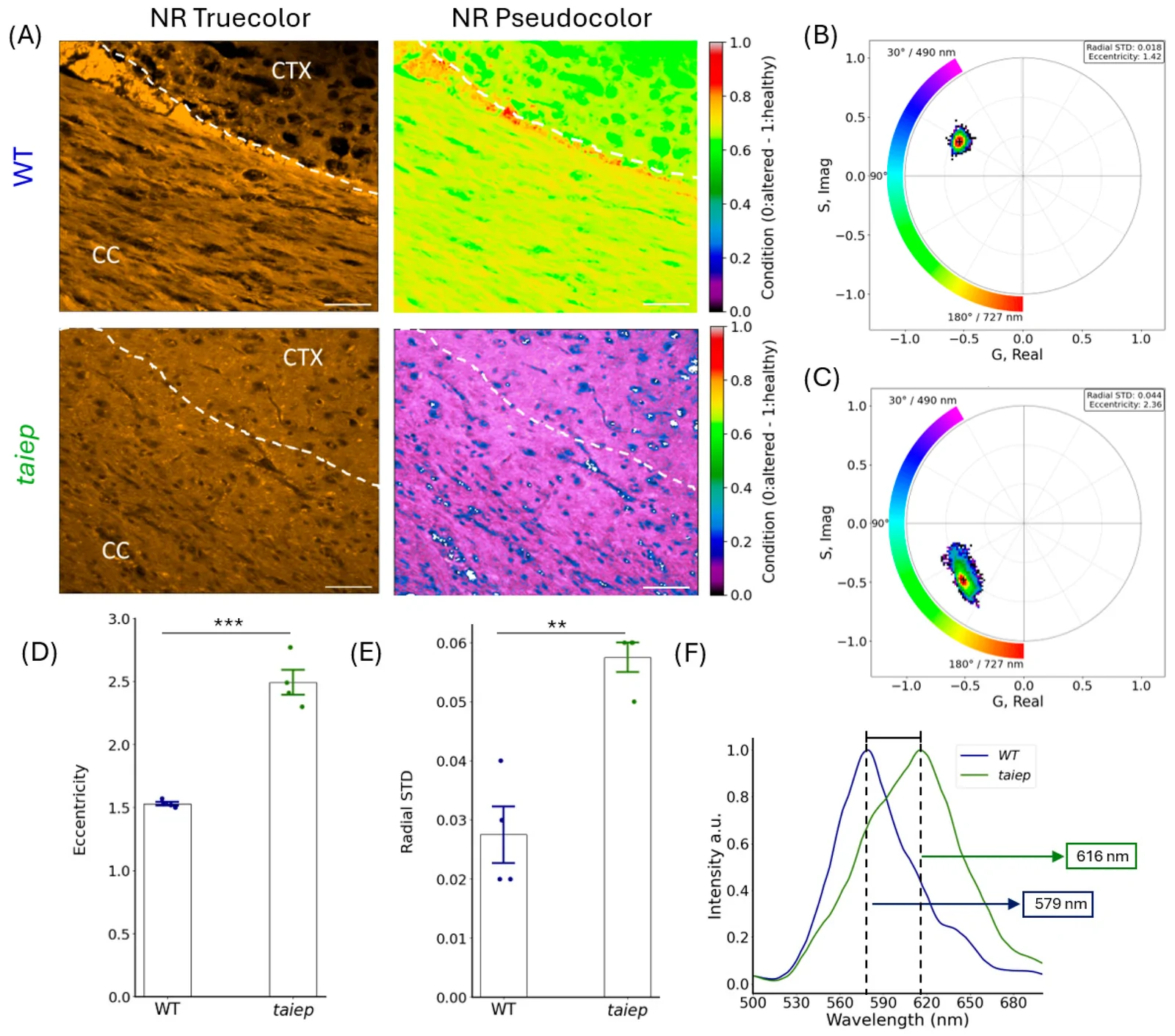
Nile Red detects myelin changes in the corpus callosum (CC) of *taiep* rats. (**A**) Representative confocal images of CC and cortex (CTX) in control (WT, top row) and *taiep* (bottom row) rats. Left panels: true-color NR fluorescence images showing general myelin staining. Right panels: pseudocolor images representing spectral shifts, where the color scale reflects the condition from 0 healthy to 1 altered. (**B**,**C**) Phasor plots illustrating the spectral distribution of NR emission in each condition. Radial standard deviation and eccentricity values are indicated. (**D**,**E**) Quantification of phasor eccentricity and radial standard deviation (mean ± SEM, ** *p* < 0.01, *** *p* < 0.001), showing significantly higher heterogeneity in *taiep* CC. (**F**). Average NR emission spectra from the CC of WT (blue) and *taiep* (green) rats, indicating a red shift in *taiep*. Scale bars: 20 µm. For each experimental condition, 80 images were analyzed, comprising 20 images from each of four animals. Image-level measurements were grouped by animal, with each animal considered the biological unit for statistical analysis. One-way ANOVA showed significant differences on radial standard deviation F(1,6) = 30.86 and eccentricity (F(1,6) = 90.63), followed by Tukey’s post hoc test.

**Figure 3:**
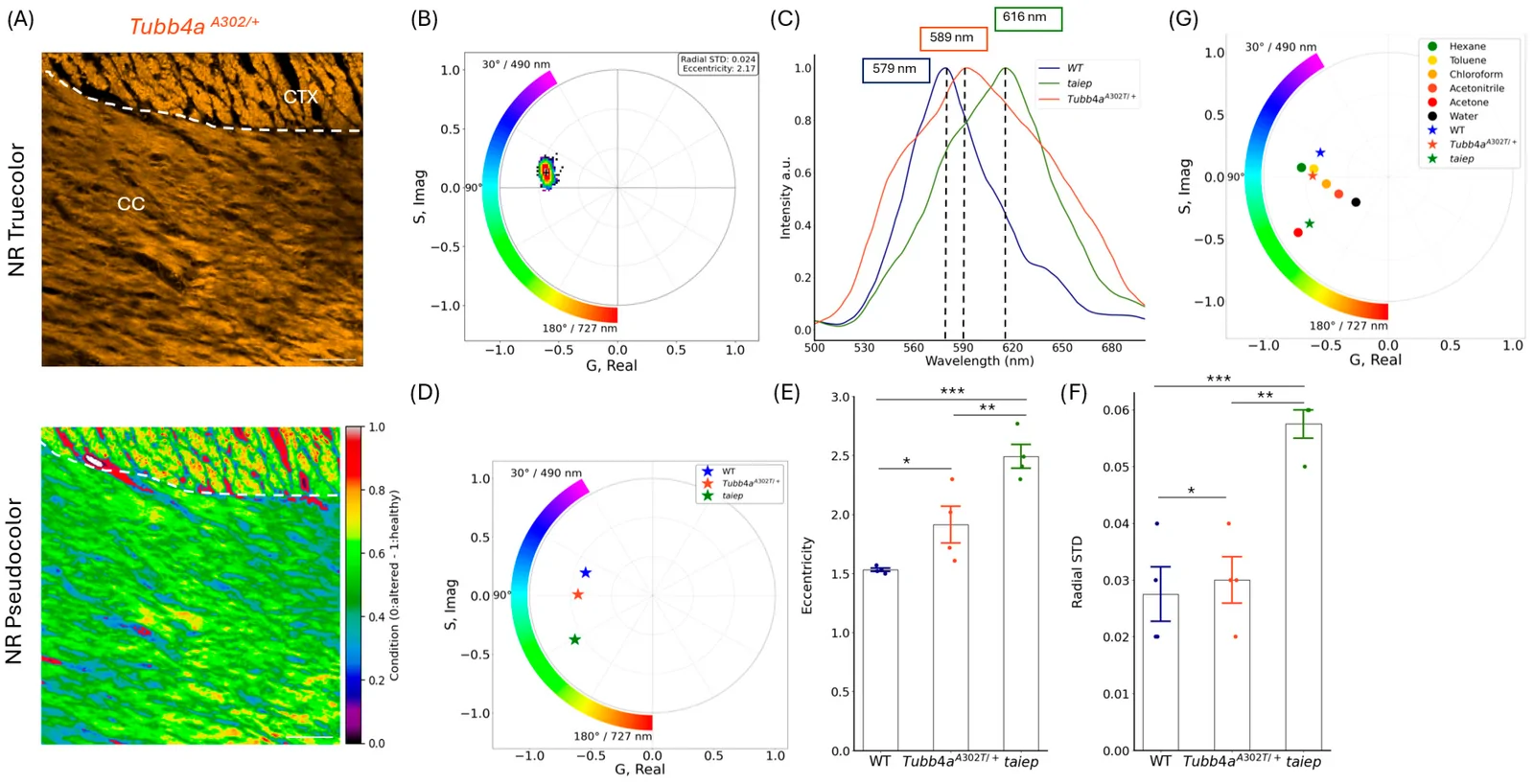
Spectral phasor analysis reveals progressive alterations in the myelin environment in heterozygous and homozygous *taiep* rats. (**A**) Representative confocal images of CC and cortex (CTX) from a heterozygous rat (*Tubb4a*^A302/+^). Top: true-color NR fluorescence images; bottom: corresponding pseudocolor representations. (**B**) Phasor plot shows the spectral distribution of NR fluorescence in the analyzed regions (representative heterozygous case illustrated in panel (**A**)). (**C**) Normalized NR emission spectra representing the mean emission profiles across all analyzed animals, showing a progressive red shift in heterozygous and *taiep* rats compared to WT, consistent with alterations in the myelin microenvironment. (**D**). Centers of mass positions in the phasor plot highlight the spectral displacement among WT, *Tubb4a*^A302/+^, and *taiep* rats. (**E**,**F**) Quantification of eccentricity (**E**) and radial standard deviation (**F**) shows significantly higher values in *Tubb4a*^A302/+^ and *taiep* rats compared to WT, reflecting increased microenvironmental heterogeneity within the myelin sheath. (**G**) Phasor plot showing tissue centers of mass (as in **D**) alongside those of reference solvents (Figure 1B). Each point corresponds to an independent experimental measurement (single case), including individual solvent acquisition and tissue samples from the three conditions (WT, heterozygous, and *taiep* rats). For each experimental condition, 80 images were analyzed, comprising 20 images from each of four animals. Image-level measurements were grouped by animal, with each animal considered the biological unit for statistical analysis. One-way ANOVA revealed a significant effect of phenotype on both radial standard deviation and eccentricity (radial standard deviation: F(2,9) = 18.14; eccentricity: F(2,9) = 20.43; * *p* < 0.05, ** *p* < 0.01, *** *p* < 0.001), followed by Tukey’s post hoc test showing significant differences between phenotypes WT, *Tubb4a*^A302/+^, and *taiep*.

## Data Availability

The data that support the findings of this study are openly available as “Myelin changes in H-ABC tubulinopathy revealed by Nile Red” at https://www.ebi.ac.uk/biostudies/bioimages/studies/S-BIAD3455 (accessed on 29 July 2026).

## Supplementary Materials

The following supporting information can be downloaded at: https://www.mdpi.com/article/10.3390/ijms27219934/s1.

## Abbreviations

H-ABC	Hypomyelination with atrophy of the basal ganglia and cerebellum
TUBB4A	Tubulin beta-4a isotype/gene
HSI	Hyperspectral imaging
NR	Nile Red
CNS	Central nervous system
MRI	Magnetic resonance imaging
PBS	Phosphate-buffered saline
PFA	Paraformaldehyde
DMSO	Dimethyl sulfoxide
CC	Corpus callosum
CTX	Cortex
WT	Wild type
FWHM	Full width at half maximum
ANOVA	Analysis of variance
SEM	Standard error of the mean
FLIM	Fluorescence lifetime imaging microscopy
HSV	Hue, saturation, value color model
LSM	Laser scanning microscope

## References

[1] Chakraborti S., Natarajan K., Curiel J., Janke C., Liu J. (2016). The emerging role of the tubulin code: From the tubulin molecule to neuronal function and disease. Cytoskeleton.

[2] Breuss M.W., Leca I., Gstrein T., Hansen A.H., Keays D.A. (2017). Tubulins and brain development—The origins of functional specification. Mol. Cell. Neurosci..

[3] Jaglin X.H., Chelly J. (2009). Tubulin-related cortical dysgeneses: Microtubule dysfunction underlying neuronal migration defects. Trends Genet..

[4] Keays D.A., Tian G., Poirier K., Huang G.-J., Siebold C., Cleak J., Oliver P.L., Fray M., Harvey R.J., Molnár Z. (2007). Mutations in alpha-tubulin cause abnormal neuronal migration in mice and lissencephaly in humans. Cell.

[5] Gonçalves F.G., Freddi T.A.L., Taranath A., Lakshmanan R., Goetti R., Feltrin F.S., Mankad K., Teixeira S.R., Hanagandi P.B., Arrigoni F. (2018). Tubulinopathies. Top. Magn. Reson. Imaging.

[6] Curiel J., Bey G.R., Takanohashi A., Bugiani M., Fu X., Wolf N.I., Nmezi B., Schiffmann R., Bugaighis M., Pierson T. (2017). TUBB4A mutations result in specific neuronal and oligodendrocytic defects that closely match clinically distinct phenotypes. Hum. Mol. Genet..

[7] Holmgren B., Urbá-Holmgren R., Riboni L., Vega-SaenzdeMiera E.C. (1989). Sprague Dawley rat mutant with tremor, ataxia, tonic immobility episodes, epilepsy and paralysis. Lab. Anim. Sci..

[8] Garduno-Robles A., Alata M., Piazza V., Cortes C., Eguibar J.R., Pantano S., Hernandez V.H. (2020). MRI Features in a Rat Model of H-ABC Tubulinopathy. Front. Neurosci..

[9] Lopez-Juarez A., Gonzalez-Vega A., Kleinert-Altamirano A., Piazza V., Garduno-Robles A., Alata M., Villaseñor-Mora C., Eguibar J.R., Cortes C., Padierna L.C. (2021). Auditory impairment in H-ABC tubulinopathy. J. Comp. Neurol..

[10] Lunn K.F., Clayton M.K., Duncan I.D. (1997). The temporal progression of the myelination defect in the taiep rat. J. Neurocytol..

[11] Alata M., González-Vega A., Piazza V., Kleinert-Altamirano A., Cortes C., Ahumada-Juárez J.C., Eguibar J.R., López-Juárez A., Hernandez V.H. (2021). Longitudinal Evaluation of Cerebellar Signs of H-ABC Tubulinopathy in a Patient and in the taiep Model. Front. Neurol..

[12] Duncan I.D., Bugiani M., Radcliff A.B., Moran J.J., Lopez-Anido C., Duong P., August B.K., Wolf N.I., van der Knaap M.S., Svaren J. (2017). A mutation in the Tubb4a gene leads to microtubule accumulation with hypomyelination and demyelination. Ann. Neurol..

[13] Eguibar J.R., Cortes C., Hernandez V.H., Lopez-Juarez A., Piazza V., Carmona D., Kleinert-Altamirano A., Morales-Campos B., Salceda E., Roncagliolo M. (2024). 4-aminopyridine improves evoked potentials and ambulation in the taiep rat: A model of hypomyelination with atrophy of basal ganglia and cerebellum. PLoS ONE.

[14] Teo W., Caprariello A.V., Morgan M.L., Luchicchi A., Schenk G.J., Joseph J.T., Geurts J.J.G., Stys P.K. (2021). Nile Red fluorescence spectroscopy reports early physicochemical changes in myelin with high sensitivity. Proc. Natl. Acad. Sci. USA.

[15] Bhargava A., Sachdeva A., Sharma K., Alsharif M.H., Uthansakul P., Uthansakul M. (2024). Hyperspectral imaging and its applications: A review. Heliyon.

[16] Schuty B., Martínez S., Guerra A., Lecumberry F., Magliano J., Malacrida L. (2023). Quantitative melanoma diagnosis using spectral phasor analysis of hyperspectral imaging from label-free slices. Front. Oncol..

[17] Teo W., Morgan M.L., Stys P.K. (2024). Quantitation of the physicochemical properties of myelin using Nile Red fluorescence spectroscopy. J. Neurochem..

[18] Sase S., Almad A.A., Boecker C.A., Guedes-Dias P., Li J.J., Takanohashi A., Patel A., McCaffrey T., Patel H., Sirdeshpande D. (2020). Tubb4a mutations result in both glial and neuronal degeneration in an H-ABC leukodystrophy mouse model. eLife.

[19] Gao F., Tom E., Skowronska-Krawczyk D. (2022). Dynamic Progress in Technological Advances to Study Lipids in Aging: Challenges and Future Directions. Front. Aging.

[20] Greenspan P., Mayer E.P., Fowler S.D. (1985). Nile red: A selective fluorescent stain for intracellular lipid droplets. J. Cell Biol..

[21] Owen D.M., Rentero C., Magenau A., Abu-Siniyeh A., Gaus K. (2012). Quantitative imaging of membrane lipid order in cells and organisms. Nat. Protoc..

[22] Valanciunaite J., Kempf E., Seki H., Danylchuk D.I., Peyriéras N., Niko Y., Klymchenko A.S. (2020). Polarity Mapping of Cells and Embryos by Improved Fluorescent Solvatochromic Pyrene Probe. Anal. Chem..

[23] O’Rourke E.J., Soukas A.A., Carr C.E., Ruvkun G. (2009). C. elegans Major Fats Are Stored in Vesicles Distinct from Lysosome-Related Organelles. Cell Metab..

[24] Sessa L., Concilio S., Di Martino M., Nardiello A.M., Miele Y., Rossi F., Brunetti J., Panunzi B., Piotto S. (2021). A selective Nile Red based solvatochromic probe: A study of fluorescence in LUVs and GUVs model membranes. Dyes Pigm..

[25] Kister A., Kister I. (2023). Overview of myelin, major myelin lipids, and myelin-associated proteins. Front. Chem..

[26] Krsulovic J., Couve E., Roncagliolo M. (1999). Dysmyelination, demyelination and reactive astrogliosis in the optic nerve of the taiep rat. Biol. Res..

[27] Jhelum P., Santos-Nogueira E., Teo W., Haumont A., Lenoël I., Stys P.K., David S. (2020). Ferroptosis Mediates Cuprizone-Induced Loss of Oligodendrocytes and Demyelination. J. Neurosci..

[28] Grochmal J., Teo W., Gambhir H., Kumar R., Stratton J.A., Dhaliwal R., Brideau C., Biernaskie J., Stys P., Midha R. (2020). Spectral Characterization of Stem Cell-Derived Myelination within the Injured Adult PNS Using the Solvatochromic Dye Nile Red. Cells.

[29] Di Giacinto F., De Angelis C., De Spirito M., Maulucci G. (2018). Quantitative imaging of membrane micropolarity in living cells and tissues by spectral phasors analysis. MethodsX.

[30] Sanchez S.A., Tricerri M.A., Gratton E. (2012). Laurdan generalized polarization fluctuations measures membrane packing micro-heterogeneity in vivo. Proc. Natl. Acad. Sci. USA.

[31] Tu K., Klein M.L., Tobias D.J. (1998). Constant-pressure molecular dynamics investigation of cholesterol effects in a dipalmitoylphosphatidylcholine bilayer. Biophys. J..

[32] Bartzokis G. (2004). Age-related myelin breakdown: A developmental model of cognitive decline and Alzheimer’s disease. Neurobiol. Aging.

[33] Wozniak J.R., Lim K.O. (2006). Advances in white matter imaging: A review of in vivo magnetic resonance methodologies and their applicability to the study of development and aging. Neurosci. Biobehav. Rev..

[34] Malacrida L., Gratton E., Jameson D.M. (2015). Model-free methods to study membrane environmental probes: A comparison of the spectral phasor and generalized polarization approaches. Methods Appl. Fluoresc..

[35] Malacrida L., Astrada S., Briva A., Bollati-Fogolín M., Gratton E., Bagatolli L.A. (2016). Spectral Phasor analysis of LAURDAN fluorescence in live A549 lung cells to study the hydration and time evolution of intracellular lamellar body-like structures. Biochim. Biophys. Acta Biomembr..

[36] GitHub—Phasorpy/Phasorpy: An Open-Source Python Library for the Analysis of Luminescence Lifetime and Hyperspectral Images Using the Phasor Approach. https://github.com/phasorpy/phasorpy.

[37] Gohlke C., Pannunzio B., Schüty B., Blanco R. (2025). “Phasorpy/Phasorpy: V0.8.”, Zenodo. https://zenodo.org/records/17316993.

