# Supplementary material for: Myelin Changes in H-ABC Tubulinopathy Revealed by Nile Red Hyperspectral Imaging
Source: Int J Mol Sci. 2026 Nov 6;27(21):9934. doi: 10.3390/ijms27219934 (PMC13763983; doi:10.3390/ijms27219934)
Supplement: Supplementary file 1 [file ijms-27-09934-s001.zip › ijms-4520487-supplementary.pdf]

Supplementary information

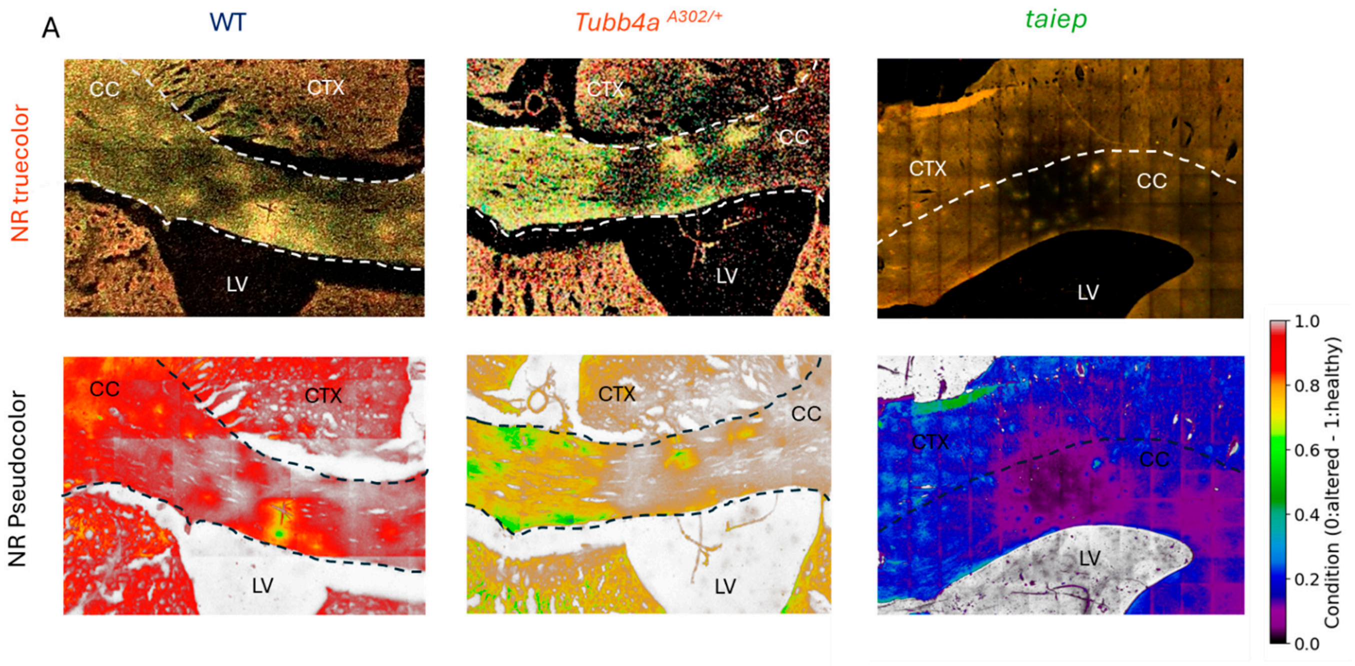

Figure S1. Comparative hyperspectral imaging of *NR* fluorescence in WT, Heterozygous, and *taiep* CC. A. Top: True-color fluorescence images of the corpus callosum stained with Nile Red from WT (left), heterozygous (middle), and *taiep* (right) samples. Bottom: Corresponding pseudocolor images generated using the spectral phasor approach, shown in the same left-to-right order (WT, heterozygous, *taiep*).
